# Anomalous Temperature Dependence of Photoluminescence Caused by Non-Equilibrium Distributed Carriers in InGaN/(In)GaN Multiple Quantum Wells

**DOI:** 10.3390/nano11041023

**Published:** 2021-04-16

**Authors:** Yuhao Ben, Feng Liang, Degang Zhao, Xiaowei Wang, Jing Yang, Zongshun Liu, Ping Chen

**Affiliations:** 1 State Key Laboratory of Integrated Optoelectronics, Institute of Semiconductors, Chinese Academy of Sciences, Beijing 100083, China; yhben@semi.ac.cn (Y.B.); wangxw@semi.ac.cn (X.W.); yangjing333@semi.ac.cn (J.Y.); zsliu@semi.ac.cn (Z.L.); pchen@semi.ac.cn (P.C.); 2 College of Materials Science and Opto-Electronic Technology, University of Chinese Academy of Sciences, Beijing 100049, China; 3 Center of Materials Science and Optoelectronics Engineering, University of Chinese Academy of Sciences, Beijing 100049, China

**Keywords:** anomalous temperature-dependent photoluminescence, non-equilibrium carriers dynamics, InGaN/GaN MQWs, hydrogen treatment

## Abstract

An increase of integrated photoluminescence (PL) intensity has been observed in a GaN-based multiple quantum wells (MQWs) sample. The integrated intensity of TDPL spectra forms an anomalous variation: it decreases from 30 to 100 K, then increases abnormally from 100 to 140 K and decreases again when temperature is beyond 140 K. The increased intensity is attributed to the electrons and holes whose distribution are spatial non-equilibrium distributed participated in the radiative recombination process and the quantum barrier layers are demonstrated to be the source of non-equilibrium distributed carriers. The temperature dependence of this kind of spatial non-equilibrium carriers’ dynamics is very different from that of equilibrium carriers, resulting in the increased emission efficiency which only occurs from 100 to 140 K. Moreover, the luminescence efficiency of MQWs with non-equilibrium carriers is much higher than that without non-equilibrium carriers, indicating the high luminescence efficiency of GaN-based LEDs may be caused by the non-equilibrium distributed carriers. Furthermore, a comparison analysis of MQWs sample with and without hydrogen treatment further demonstrates that the better quantum well is one of the key factors of this anomalous phenomenon.

## 1. Introduction

In the past two decades, GaN based materials are widely applied in optoelectronics devices including light emitting diodes (LED) and lasers which have got great success in display and general illumination [1,2,3,4,5,6,7]. One of the attractive features of GaN-based materials is the high luminous efficiency of the InGaN/(In)GaN multiple quantum wells (MQWs) even when the dislocation density is as high as 10^8^–10^9^ cm^−2^ [8]. To understand the carrier recombination mechanism in GaN-based MQWs, temperature-dependent photoluminescence (TDPL) spectra have been widely used for the investigation of optical properties in GaN-based MQWs samples [9,10,11,12,13]. On one hand, the shift of emission peak energy of the photoluminescence spectra with increasing temperature indicates the carrier transport process between different localization states [9,14,15,16]. On the other hand, the decline of photoluminescence intensity with increasing temperature demonstrates the thermal activation of non-radiative recombination centers [17] and the process of carrier hopping out of localization states [18,19]. Based on the above -mentioned reports, the localized states are widely accepted to be responsible for the high luminous efficiency of GaN MQWs. However, some aspects of the luminescence mechanism remain ambiguous. For example, some researchers indicates that the non-equilibrium distribution of electrons and holes, which is caused by the large polarization electric field, plays an important role in the luminescence of the localized states [20].

In this work, the mechanism of high luminescence efficiency of GaN MQWs are further investigated based on the analysis of an anomalous increase of the integrated PL intensity with increasing temperature. Such improved emission efficiency can be attributed to the increasing probability of overlap in space of the non-equilibrium distributed electrons and holes which are transported from quantum barriers. The results indicate that the high luminous efficiency of the InGaN/(In)GaN multiple quantum wells (MQWs) LEDs may origin from the recombination process of the non-equilibrium distributed electrons and holes.

## 2. Materials and Methods

Two InGaN/(In)GaN MQW samples, named A and B, were grown on c-plane sapphire substrate by a Thomas Swan 3 × 2 in. close-coupled showerhead reactor MOCVD. During the epitaxial growth process, the triethylgallium (TEGa), trimethylindium (TMIn) and ammonia (NH_3_) were used as precursors for Ga, In and N sources, respectively. Both of the samples consist of a 2 μm thick Si-doped GaN layer, a two-period unintentionally doped InGaN/(In)GaN MQWs active region and a 150 nm Mg-doped GaN layer. During the epitaxial growth of the MQWs at 750 °C, a thin GaN cap layer was grown after the growth of the InGaN well, then a (In)GaN barrier layer was deposited on the cap layer. For sample A, a 100 s hydrogen (H_2_) treatment with 200 sccm H_2_ was conducted after the growth of the cap layer. However, for the sample B, after the growth of the cap layer, the (In)GaN barrier was immediately grown. However, other than that, the growth conditions of the two samples were totally identical.

Temperature-dependent (TD) photoluminescence (PL) spectra, which were recorded between 30 K and 300 K, were measured using a 325 nm laser and a 405 nm laser in a closed-cycle helium refrigerator of CTI Cryogenics. The samples are measured under same conditions and the spectrometer has been corrected for the wavelength dependence of the detector response. Meanwhile, microscopic photoluminescence (μ-PL) was performed to characterize the luminescence properties of the MQW samples in a microscopic scale using a Nikon A1 confocal optical system (Nikon, Japan) excited with a 405 nm laser.

## 3. Results and Discussion

**Figure 1:**
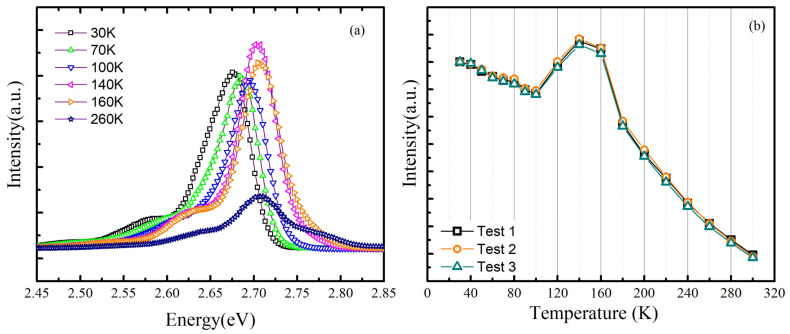
(**a**) The photoluminescence spectra at typical temperature of sample A; and (**b**) the normalized temperature-dependent integrated Photoluminescenceintensity of three repeated tests of sample A.

Firstly, the TDPL spectra from 30 to 300 K of sample A has been measured with a 325 nm laser as excitation source. Figure 1a shows the measured PL spectra at different typical temperature. Note that the luminescence intensity increases intensely from 100 to 140 K, which is much different from the conventional TDPL spectra. In order to make quantitative analysis on the integrated intensity, Figure 1b shows the integrated PL intensity of three repeated tests of sample A, which can eliminate the contingency caused by experimental error (only the results of test 1 are discussed in the next sections due to the repeatability). The integrated PL intensity of sample A forms an anomalous variation with increasing temperature: first, it decreases and then increases anomalously from 100 to 140 K and, finally, declines rapidly when the temperature rises beyond 140 K. To exclude the random mistakes caused by the equipment and operation, we have repeated three times and got same results. Thus, it is safe to say the results are real and credible. In fact, a luminescence behavior called “thermal quenching” usually emerges in the normal TDPL spectra [9,21] and the intensity will monotonically decrease. At very low temperature, the internal quantum efficiency of MQWs luminescence is often taken as almost 100%, considering that the non-radiative recombination centers are strongly suppressed. When temperature increases, the non-radiative recombination centers are thermally activated, capturing more photo-generated carriers and decreasing the integrated PL intensity. In other words, the number of carriers participated in the radiative recombination process will decrease with increasing temperature. However, in sample A, the integral PL intensity has an anomalous increase from 100 to 140 K. It indicates that there must be extra carriers injected into the radiative recombination centers, enhancing the integral PL intensity [22]. Therefore, it is necessary to find the source of the extra carriers first.

**Figure 2:**
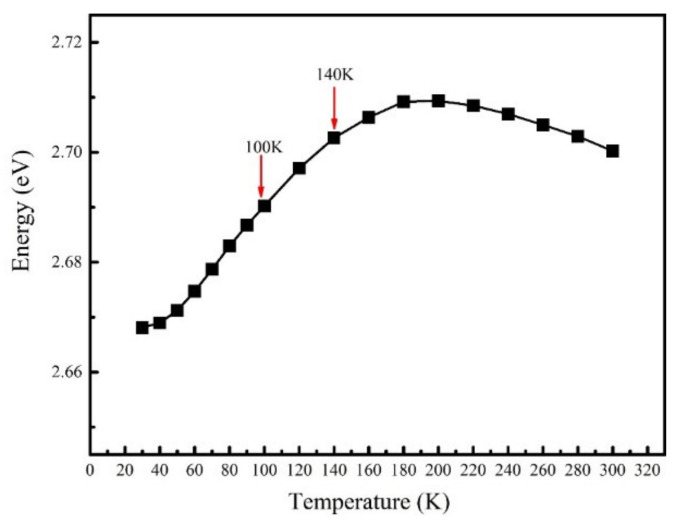
The PL emission peak energy as a function of temperature for sample A.

One possible explanation is that the extra carriers are transferred from shallower localized potential centers to deeper localized potential centers [20,21]. It is well known that deeper localized potential traps can screen dislocation and other defects more effectively, improving the carrier radiative recombination efficiency. Note that this explanation is based on the carrier transfer process from shallower traps to deeper traps, which means the red shift of the PL emission peak [14]. To check that, the curves of PL emission peak energy as a function of temperature for samples A are shown in Figure 2. It can be found that, contrary to the redshift, the peak energy has a significant blue shift from 2.69 to 2.70 eV between 100 and 140 K, which means a transfer process of carriers from deeper traps to shallow traps. Such a contradiction suggests that the transfer process may not be the main reason to this intensity enhancement phenomenon. In addition, the trend of blue shift is slowing down after 160 K, which is related to anomalous increase of luminescence intensity, which will be discussed below.

**Figure 3:**
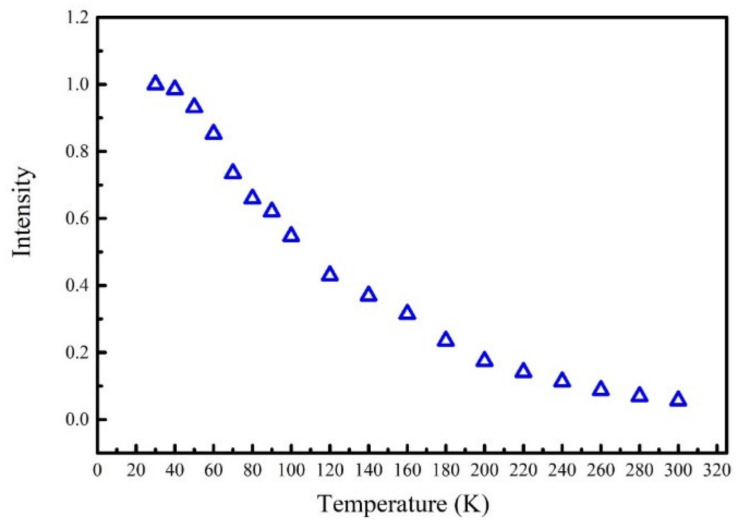
The normalized integrated PL intensity measured with 405 nm laser of sample A.

Another more reasonable interpretation is that the extra carriers come from the quantum barriers (QBs) since a 325 nm laser is used as the excitation source and a similar phenomenon has been observed in previous work [22]. The photon energy of 325 nm laser is higher than the barrier band gap; thus, extra carriers can be excited in the barrier layers and then dropped into quantum wells. A simple but effective method is used to confirm that the extra carriers come from the QBs. The TDPL spectra from 30 to 300 K has been measured using a 405 nm laser as excitation light source. As is well known, the excitation energy of 405 nm laser is lower than the quantum barrier gap. It cannot excite carriers in quantum barrier layers. The integral intensity of PL spectra measured with 405 nm laser excitation are shown in Figure 3. It is found that, contrary to the anomalous increase intensity of the PL spectra measured with 325 nm laser shown in Figure 1b, a typical “thermal quenching” behavior appears in the PL spectra measured with 405 nm laser. The result demonstrates that the extra carriers participated in the radiative recombination process come from QB layers indeed. In addition, the results also indicate that the contribution of carriers generated in quantum wells (W-carriers) decreases monotonously with increase of temperature.

Based on the above discussion, both the carriers generated in quantum barriers (B-carriers) and W-carriers contribute to the PL measured with a 325 nm laser. However, for the TDPL spectra measured with a 405 nm laser, only the W-carriers contribute to the radiative recombination process. Therefore, by comparing the normalized integrated PL intensity measured with 325 nm laser and 405 nm laser, one can effectively exclude the effect of W-carriers on the luminescence and gain a deeper insight of the effect of B-carrier on luminescence.

**Figure 4:**
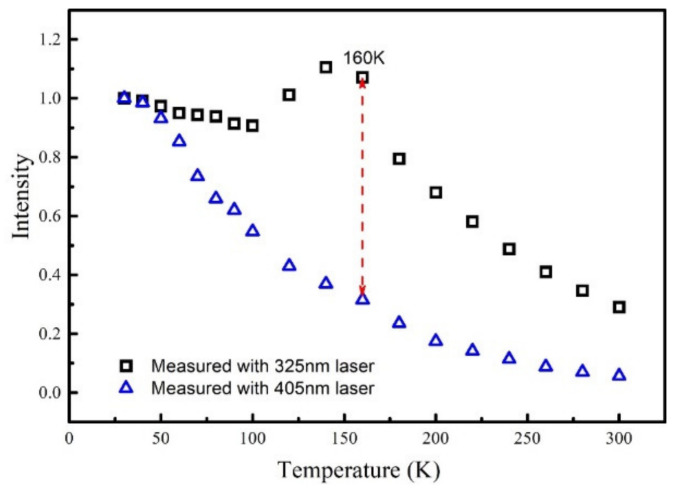
The comparison of the normalized integrated PL intensity measured with a 325 nm laser and a 405 nm laser for sample A.

Figure 4 shows the normalized integrated PL intensity excited by 325 and 405 nm laser of sample A. In fact, the normalized integrated PL intensity are widely accepted to represent the luminescence efficiency at different temperature assuming that the non-radiative recombination centers are totally suppressed at low temperature [12,23]. It is found that, at low temperature, e.g., 30 to 50 K, the B-carriers have little contribution to the luminescence. When temperature is beyond 50 K, the values of the normalized PL intensity measured with a 325 nm laser are far more than that measured with a 405 nm laser. Furthermore, the difference of the normalized integrated PL intensity measured by 325 nm laser and 405 nm laser increases between 50–160 K, showing that the contribution of B-carriers reaches highest point when temperature is 160 K. However, as temperature rises further, the gap between the two is gradually narrowing, meaning that the contribution of B-carriers decreases. In summary, the contribution of B-carriers to the luminescence increases firstly and then decreases with increasing temperature.

**Figure 5:**
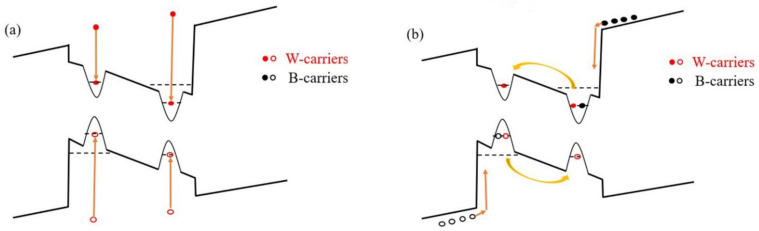
(**a**): The schematic diagram of the energy band of the quantum well and equilibrium distributed W-carriers; (**b**): the transport process of B-carriers whose distribution is non-equilibrium in space. The yellow arrows represent the carrier transport and relaxation process.

From the above results, the temperature dependence of the contribution of W-carrier and B-carrier to the luminescence is very different. Figure 5 shows the schematic diagram of the energy band of the quantum well and different radiative recombination process of W-carriers (In Figure 5a) and B-carriers (In Figure 5b). The interface charges caused by the mismatch of lattice between InGaN wells and GaN barriers lead to strong polarization electric field across the QW layers, resulting the tilt of energy band as shown in Figure 5. However, in the InGaN/GaN MQWs samples, the localized potential traps can effectively screen the effect of polarization electric field [24]; thus, the carriers excited randomly in quantum wells (W-carriers) will distribute in the localized potential traps across the whole quantum well at very low temperature, as shown in Figure 5a. When temperature increases, the W-carriers will hop out of the localized potential traps, resulting in a monotonous decrease of radiative recombination process [12]. However, under the effect of built-in electric field, electrons and holes of B-carriers are drift from different direction into the quantum well and captured by the localized potential centers, resulting in a spatial separation of electron-hole pairs at the low temperature [20], as shown in Figure 5b. In other word, the distribution of the carriers transported from the QB layers is non-equilibrium in space. The non-equilibrium carriers transported into the quantum well layers show a difference radiative recombination path compared to the W-carriers. When the temperature is very low, e.g., 30 to 50 K, the carriers do not have enough kinetic energy to hop out of the potential traps and overcome the polarization electric field, so the overlap integral of the wavefunctions of electrons and holes is very low. As a result, the B-carriers has little contribution to the luminescence at low temperature range. As the temperature rises from 50 to 160 K, the carriers become hotter and will occupy higher energy levels, they are more possible to hop out of the potential traps and overcome the polarization electric field, which is confirmed by the blue shift process of the PL peak as shown in Figure 2. The wavefunctions of more electrons and holes overlap and the radiative recombination probability is strongly improved. As the temperature increases further, e.g., beyond 160 K, the thermal energy of carriers is high enough to overcome the whole energy barrier of traps, which is disclosed by the red shift of the emission peak energy as shown in Figure 2. At the same time, the non-radiative recombination centers are activated further at high temperature range. As a result, most of the B-carriers will be captured by the non-radiative recombination centers rather than the localized potential traps; thus, the effect of non-equilibrium carriers on luminescence drops rapidly.

The S-shape of luminescence intensity of TDPL measured with 325 nm laser actually reflects the competition process between the luminescence increase due to non-equilibrium carriers and the luminescence loss caused by the thermal activation of non-radiation recombination centers. At the temperature range of 30–100 K, the contribution of non-equilibrium carriers to luminescence cannot offset all the luminescence loss; thus, the luminescence intensity shows a slight decrease. As the temperature rises from 100 to 140 K, the luminescence increase caused by non-equilibrium carriers is far more than the luminescence loss; thus, the luminescence intensity increases strongly. While temperature is beyond 140 K, non-radiative recombination process dominates the recombination process, resulting in a drop of intensity.

From Figure 4, it is also found that the luminescence efficiency of the MQWs with non-equilibrium carriers participated is much higher than that without non-equilibrium carriers. When the temperature is 300 K, the luminescence efficiency of the MQWs with non-equilibrium carriers participated is around 30%, while the luminescence efficiency of the MQWs without non-equilibrium carriers is around 8%. One of the attractive features of GaN-based materials is the high luminous efficiency of the InGaN/(In)GaN LEDs even when the dislocation density is as high as 10^8^–10^9^ cm^−2^ [8]. Considering that the electrons and holes are non-equilibrium distributed in QWs under the condition of electric injection, the high luminous efficiency is closely related to the transported carriers. To further investigate the effect of transported carriers on the luminescence, another sample (Sample B) without hydrogen annealing treatment on the QW layers is prepared, considering that the hydrogen annealing treatment can effectively etch the In-rich layers and improve the interface quality of the MQW [25,26] which can provide more non-equilibrium carriers participated in luminescence.

**Figure 6:**
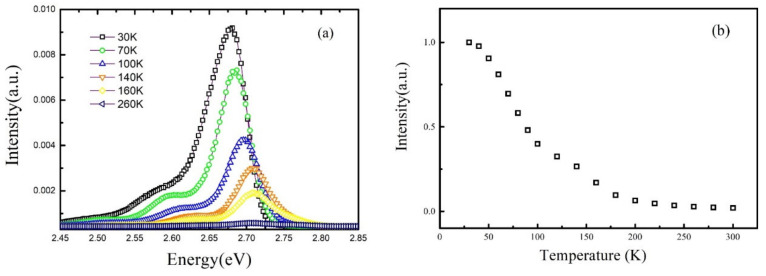
(**a**): The photoluminescence spectra of sample B at different temperature; (**b**): the normalized temperature-dependent integrated PL intensity of sample B.

Figure 6 shows the temperature dependence of the normalized integrated PL intensity measured with 325 nm laser excitation for sample B. It is found that this anomalous increase of integrated PL intensity with increasing temperature does not appear in the sample B and the luminescence efficiency at 300 K is around 5%, which is much lower than that of sample A. On one hand, the result indicates that this abnormal increase of integrated intensity only appears in the MQWs samples with less non-radiative recombination centers. On the other hand, the contribution of non-equilibrium carriers to the luminescence is low when the MQWs has a worse quality. Based on that, it is easy to interpret the luminescence behavior of sample B. On one side, the non-radiative centers in the QWs can capture more carriers transported from QBs; thus, less carriers can get into the localized potential traps in quantum wells. On the other side, as the temperature rises, instead of reaching the radiative recombination centers at the same position in space after hopping out of the located potential traps, the non-equilibrium distributed electrons and holes are more possible to be captured by the non-radiative recombination center.

**Figure 7:**
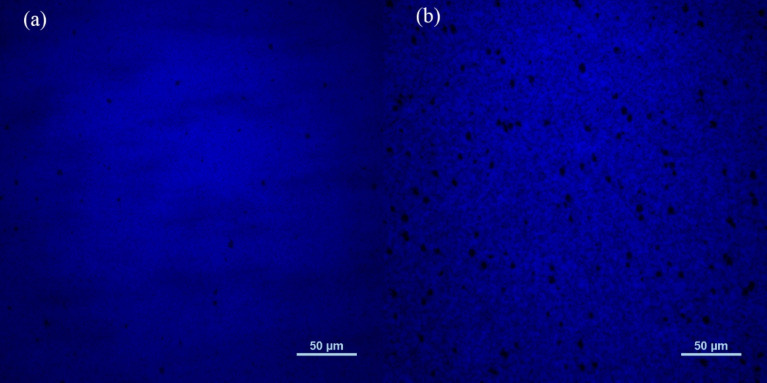
Comparison of μ-PL results between sample A (**a**) and B (**b**).

Figure 7 shows a comparison of μ-PL results of the sample A and B at room temperature. It is found that the average size and number of non-luminous regions of sample B are significantly larger than that of the sample A, indicating a stronger non-radiative recombination process for sample B. It is well known that indium atoms tend to accumulate around dislocation defects, resulting in a strong restriction effect on carriers around the area. Meanwhile, the dislocation defects generally produce V-shape pits on the QBs [27], in which the carriers are more possible to participate the non-radiative recombination process. With H_2_-treatment on the surface of the quantum wells, the adatom indium in the InGaN epitaxial layer will be removed by the hydrogen atom due to the etching effect [25,28,29], weakening the restriction effect on carriers. As a result, the MQWs of sample A is much better than that of sample B, leading to a higher luminescence efficiency of sample A.

## 4. Conclusions

In summary, an anomalous increase of integrated TDPL intensity has been observed between 100 K and 140 K in a GaN-based MQWs sample. This phenomenon is ascribed to the enhanced radiative recombination process caused by transported carrier dynamics carriers excited in the QBs. To interpret that the increase of luminescence intensity only occurs between 100 K and 140 K, a temperature-dependent carrier dynamics model is proposed, which is related to the overlap in space of the non-equilibrium distributed carriers. Moreover, the results indicate that the high luminous efficiency of the InGaN/(In)GaN multiple quantum wells (MQWs) LEDs may origin from the recombination process of the non-equilibrium electrons and holes.

## Data Availability

The data that support the findings of this study are available from the corresponding author upon reasonable request.
